# Transcriptomic and Lipidomic Analysis Reveals the Regulatory Network of Lipid Metabolism in *Cannabis sativa*

**DOI:** 10.3390/foods14162809

**Published:** 2025-08-13

**Authors:** Bowei Yan, Chuanyi Chang, Yue Sui, Nan Zheng, Yuyan Fang, Yuanye Zhang, Ming Zhang, Dan He, Liguo Zhang

**Affiliations:** 1 Institute of Industrial Crops, Heilongjiang Academy of Agricultural Sciences, Harbin 150086, China; yanbowei21@outlook.com (B.Y.); hljnkysy@126.com (Y.S.); tgznhlj@163.com (N.Z.); fyytghlj@163.com (Y.F.); hljwangguijiang@163.com (Y.Z.); hljgyntg@163.com (M.Z.); 2 Harbin Academy of Agricultural Science, Harbin 150028, China; tgccyhlj@163.com; 3 Heilongjiang Academy of Land Reclamation Sciences, Harbin 150038, China; zhaochangjianghlj@163.com

**Keywords:** *Cannabis sativa* L., lipid metabolic, lipidomic, transcriptomic, regulatory network

## Abstract

Hemp possesses significant healthcare value due to its rich composition of unsaturated fatty acids and a distinctive golden ratio of linoleic acid to α-linolenic acid. As a promising special-oil crop, it holds substantial potential for development and utilization. However, the regulatory mechanisms underlying its lipid metabolic pathways remain poorly understood. In this study, the independently bred hemp seed variety Longdama No. 9 was used to construct a regulatory network of the fatty-acid and lipid metabolisms through integrative transcriptomic and lipidomic analysis. Transcriptomic profiling revealed differentially expressed genes (DEGs) involved in lipid biosynthesis across various tissues. In leaves, DEGs associated with glycerolipid synthesis were generally upregulated compared to in roots and seeds. In seeds, DEGs involved in fatty-acid synthesis and triacylglycerol (TAG) assembly were predominantly upregulated. Meanwhile, root tissues showed a higher abundance of upregulated DEGs related to phospholipid biosynthesis. Lipidomic analysis further highlighted tissue-specific lipid distributions. The galactolipid monogalactosyldiacylglycerol (MGDG) was most abundant in the leaves. While phosphatidylglycerol (PG) had the highest molar percentage in the seeds, most other major phospholipids were predominantly found in the roots. The prevalence of the C36:6 molecular species in the MGDG and digalactosyldiacylglycerol (DGDG) indicates that hemp is a typical 18:3 plant. The combined transcriptomic and lipidomic analysis revealed that tissue-specific transcriptional regulation contributes to the unique lipid profile of hemp. These findings provide valuable insights into the regulation of lipid metabolism in hemp and identify key genes involved in oil biosynthesis, which can lay a theoretical foundation for the development and utilization of hemp as a special-oil crop.

## 1. Introduction

Hemp (*Cannabis sativa* L.) refers to cannabis cultivars in which the tetrahydrocannabinol (THC) content in the dry matter of the upper leaves and inflorescences during flowering remains below 0.3%, thereby eliminating its suitability for direct drug use [1] Currently, it is currently cultivated on a commercial scale across Canada, the United States, and China. As a high-yield, low-input, and environmentally benign crop, it serves multiple supply chains, including food, pharmaceuticals, construction, and specialty chemicals [2] Among the oldest domesticated plant species, it integrates substantial economic returns with pronounced pharmacological potential. The therapeutic relevance of hemp is primarily attributed to cannabinoids, which are biosynthesized and sequestered within the glandular trichomes of female inflorescences. These phytocannabinoids act as exogenous ligands of the endocannabinoid system, thereby modulating metabolic, immune, and neurophysiological homeostasis [3,4]. Concomitantly, they exhibit well-documented antibacterial, anti-inflammatory, and anxiolytic properties [5]. Collectively, industrial hemp represents a paradigmatic, sustainable cash crop whose versatile applications in agriculture and manufacturing are increasingly recognized as a catalyst for resilient economic growth on a global scale.

In recent years, the escalating scientific and consumer recognition of the superior nutritional profile and associated health benefits inherent to hemp seeds has positioned them as a high-value commodity with significant agronomic and commercial development potential [6]. Among all hemp tissues, only the seeds are currently permitted for food use, as they are intrinsically devoid of cannabinoids; any trace cannabinoid residues present result exclusively from adventitious, mechanically induced contamination during harvesting and downstream processing. Hemp seeds contain 25–35% lipids, with a distinctive fatty-acid profile comprising 70–90% polyunsaturated fatty acids (PUFAs) [7]. Among these, linoleic acid (18:2, n-6; LA) is the most abundant, representing more than half of the total fatty-acid content, followed by α-linolenic acid (18:3, n-3; ALA) [8]. As a rich dietary source of these two essential fatty acids (EFAs), which cannot be synthesized by the human body and must be obtained through the diet, hemp seed oil offers considerable nutritional benefits. This makes hemp a promising candidate for high-quality edible oil production, with significant potential for development and utilization [2,9].

Triacylglycerol (TAG) is the primary constituent of vegetable oils and serves as the main storage form of carbon and energy in plants. It is predominantly accumulated in specific tissues and plays a vital role in seedling development, lipid homeostasis, and the reproductive processes [10]. Fatty acids form the fundamental building blocks for TAG synthesis. In plants, TAG biosynthesis involves two major stages: fatty-acid synthesis within plastids and subsequent TAG assembly in the endoplasmic reticulum (ER) [11]. While the specific TAG biosynthetic pathways may vary across species, it is estimated that over 90% of seed fatty acids are funneled into TAG via PC intermediates [12]. Otherwise, the cell membrane is primarily composed of three major classes of lipids: glycerolipids, sphingolipids, and sterols [13]. Among these, glycerolipids serve as the principal structural components, with variations in the fatty acyl chain composition and ratios across different membrane systems. Phospholipid biosynthesis mainly occurs in the endoplasmic reticulum (ER), and common plant phospholipids include phosphatidic acid (PA), PC, phosphatidylethanolamine (PE), phosphatidylserine (PS), phosphatidylglycerol (PG), and phosphatidylinositol (PI) [14]. Glycolipids in plants exhibit structures similar to phospholipids and are categorized, based on their saccharide head groups, into monogalactosyldiacylglycerol (MGDG), digalactosyldiacylglycerol (DGDG), and sulfoquinovosyldiacylglycerol (SQDG) [15,16]. MGDG and DGDG are predominant in photosynthetic tissues and are unique to photosynthetic membranes. Alongside PG, they are essential components of the plasma membrane and play a critical role in plant photosynthesis [17]. Phospholipids are transported from the ER to chloroplasts, where they provide diacylglycerol (DAG) precursors for glycolipid biosynthesis. The composition of membrane lipids is intricately linked to the lipid synthesis, transport, turnover, remodeling, and degradation processes [18].

While the biosynthetic pathways of plant oils and associated gene functions have been extensively characterized in many higher plants, particularly in conventional oil crops [19], genetic resources and functional studies in hemp remain limited. Gene-editing strategies targeting fatty-acid composition and oil content have become central to breeding elite oilseed germplasms. In recent years, emerging special-oil crops such as hemp have gained attention as valuable biological resources for identifying key genes involved in oil biosynthesis and metabolic improvement [20,21]. Meanwhile, although interest in cannabis-derived foods intended for human consumption is intensifying, specific regulation concerning the analytical parameters for the quality assessment of hemp seed oils is still lacking [22]. However, due to strict regulatory oversight and its relatively recent industrialization, hemp still lacks comprehensive genetic information. To address this gap, the present study employed transcriptomic and lipidomic analysis to investigate the lipid-metabolism pathways of hemp and identify critical genes involved in oil biosynthesis. These findings aim to establish a foundation for deeper insights into the regulatory mechanisms and functional genomics of lipid metabolism in hemp. These findings will facilitate the targeted enhancement of lipid profiles in hemp, substantially elevate seed oil content, and furnish valuable genetic resources for the development of specialty oil crops. Furthermore, these studies could contribute to standardizing hemp seed oil quality.

## 2. Materials and Methods

### 2.1. Plant Materials and Growth Conditions

The experimental material used in this study was Longdama No. 9, a seed-type hemp variety independently developed by the Institute of Industrial Crops, Heilongjiang Academy of Agricultural Sciences (Harbin). Plants were cultivated in a greenhouse under controlled conditions: a 14 h light/10 h dark photoperiod, day/night temperatures of 22 °C/18 °C, light intensity of 20,000 lux, and relative humidity maintained at 70 ± 1% [23]. A total of 120 pots of plants under the same culture conditions were cultivated. Tissue samples were collected from leaves and roots six weeks after emergence and from seeds 14 days post-flowering during the developmental stage. Each sample set had 3 biological replicates; each replicate was made of three mixed samples. The collected samples were immediately sealed in cryogenic tubes, flash-frozen in liquid nitrogen, and stored at −80 °C until further analysis.

### 2.2. RNA Sequencing and Quantitative Real-Time PCR (qRT-PCR)

Total RNA was extracted from hemp tissues using the TRIzol reagent. RNA quality was assessed via agarose gel electrophoresis and an ultra-micro-UV-visible spectrophotometer (Thermo Fisher Scientific, Waltham, MA, USA), ensuring OD260/280 values between 1.8 and 2.0. Qualified RNA samples were used for cDNA library construction and subsequently sequenced on the Illumina HiSeq 4000 high-throughput sequencing platform. DEGs were annotated by aligning them against public databases, including Swiss-Prot, KEGG, KOG, and Pfam, to identify homologous protein sequences and assign functional annotations. Gene expression levels were quantified using the featureCounts tool (version 2.0.3), and the FPKM (fragments per kilobase of transcript per million mapped reads) method was employed to estimate transcript abundance. Differential expression analysis between various hemp tissues was conducted using the DESeq2 package, with *p*-value adjustment via the Benjamini–Hochberg method. DEGs were defined based on these criteria: Padj < 0.05 and |log2 (Fold Change) | ≥ 1. Enrichment analyses for Gene Ontology (GO) terms and KEGG pathways were then performed on the identified DEGs. To validate the RNA-seq data, twelve DEGs were selected for quantitative real-time PCR (qRT-PCR) analysis. Gene-specific primers were designed using Primer6 software (Table S1). Reverse transcription was carried out using the SuperScript IV First-Strand Synthesis System (Thermo Fisher Scientific, Cat. No. 18090050) according to the manufacturer’s instructions. qRT-PCR was performed using TOYOBO’s real-time quantitative detection kit (Cat. No. QKD-201), with Actin used as the internal reference gene. Relative expression levels were calculated using the 2^−ΔΔCt^ method.

### 2.3. Lipid Extraction and Identification

The hemp lipid extraction was slightly modified based on previous studies [24]. Briefly, tissue samples from different parts of the hemp plant were immersed in preheated isopropanol containing 0.01% butylated hydroxytoluene (BHT) at 75 °C for 15 min. Following this, 1.5 mL of chloroform and 0.6 mL of distilled water were added to each sample. The mixture was thoroughly vortexed and subjected to shaking extraction at 22 °C for 1 h at 180 rpm. After centrifugation, the supernatant was transferred to a clean tube, and 4 mL of a chloroform:methanol mixture (2:1, *v*/*v*) containing 0.01% BHT was added. The mixture was then vortexed and extracted at 22 °C for 30 min at 180 rpm. This extraction process was repeated three to four times. The combined extracts were washed with 1 mL of 1 M KCl, and the supernatant was discarded following centrifugation. Subsequently, 2 mL of distilled water was added and vortexed thoroughly, and the supernatant was again removed. The final lipid extract was dried under a stream of nitrogen gas and stored at –20 °C for later use.

The Kansas Lipidomic Research Center (KLRC, Manhattan, KS, USA) was commissioned to perform a comprehensive lipidomic analysis by electrospray ionization tandem mass spectrometry (ESI-MS/MS). This approach precisely quantified fluctuations in lipid metabolite levels while elucidating fatty-acid composition and structure. The profiles of the lipids were systematically drawn using precursor (Prec) and neutral loss (NL) strategies. The sample was introduced into the electrospray ionization source. A series of peaks in the lipid content were detected by electrospray ionization. The peaks on the spectrum were quantitatively compared with a set of internal standards. Data for each lipid molecular species were normalized and displayed as the mole percent of the total lipid analyzed.

### 2.4. Statistical Analysis and Data Visualization

Statistical analyses and graph generation were conducted using GraphPad Prism 9.0 software. Results are presented as means ± standard deviation (SD). Construction of the regulatory network diagrams was carried out using a combination of TBtools-II (v 2.315) and Microsoft Office PowerPoint 2016.

## 3. Results

### 3.1. Transcriptomic Analysis of Hemp

To elucidate the transcriptional regulatory mechanisms of the genes involved in lipid metabolism in hemp, RNA-seq analysis was conducted on nine samples derived from developing seeds, leaves, and root tissues. The raw sequencing data have been deposited in the NCBI database in FASTQ format under BioProject accession number PRJNA899681. After removing redundant sequences, a total of 60.7 Gb of clean data was obtained. The clean data per sample ranged from 6.59 to 7.37 Gb, with an average sequencing error rate of approximately 0.1% and alignment rates exceeding 85.78%. The proportion of uniquely mapped reads ranged from 81.58% to 86.61%, with Q30 base percentages exceeding 94.41% and GC content ranging between 43.03% and 44.43% (Table S2). These metrics indicate high-quality sequencing data suitable for downstream analysis. The reliability of the RNA-seq results was further validated through qRT-PCR analysis (Figure S1).

### 3.2. The GO and KEGG Enrichment Analysis

**Figure 1:**
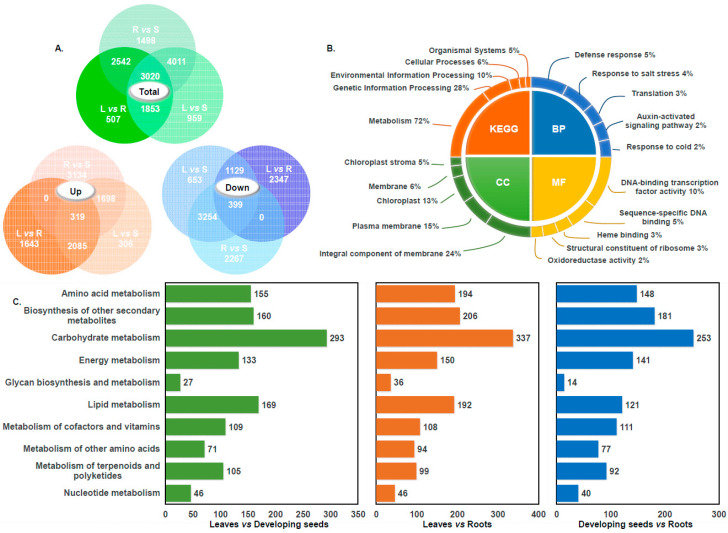
Quantitative statistics of differential genes and analysis of transcription data annotation results. (**A**) Venn diagrams illustrating the distribution of DEGs across three tissue types: leaf (L), root (R), and developing seed (S). The green diagram represents the total DEGs, the orange diagram highlights upregulated DEGs, and the blue diagram shows downregulated DEGs. (**B**) Functional classification of DEGs based on GO and KEGG pathway annotations. The bar charts display the top five terms in each GO category (biological process, cellular component, molecular function) and KEGG pathways. (**C**) DEGs involved in metabolic pathways, with different colors representing different tissue comparison groups.

DEGs in three tissues, leaves (L), roots (R), and developing seeds (S) were screened and compared (Figure 1A). The comparison between seeds and roots (S vs. R) revealed the highest number of DEGs, totaling 11,071, comprising 5151 upregulated and 5920 downregulated genes, with downregulated genes being more predominant. In contrast, the leaf vs. root (L vs. R) comparison showed the fewest DEGs, with 7922 genes identified: 4047 upregulated and 3875 downregulated. In the seed vs. leaf (S vs. L) group, a total of 9843 DEGs were detected, including 4408 upregulated and 5435 downregulated genes, with the downregulated genes outnumbering the upregulated ones.

Transcriptome data were aligned to the KEGG database, resulting in the annotation of 3130 DEGs across 125 metabolic pathways. It is noteworthy that some DEGs were involved in multiple pathways. Among these, the “Metabolism” category accounted for the majority, with 2252 DEGs (72%), followed by “Genetic Information Processing,” comprising 28%. Additional pathway categories included “Environmental Information Processing” (10%, 305 genes), “Cellular Processes” (6%, 196 genes), and “Organismal Systems” (5%, 166 genes) (Figure 1B). Among the metabolism pathways, the top five pathways with the most annotated genes were carbohydrate metabolism, amino acid metabolism, lipid metabolism, biosynthesis of other secondary metabolites, and energy metabolism pathways, accounting for 28% (508), 17% (305), 16% (292), 15% (271), and 12% (219), respectively (Table S3). A similar distribution of metabolic pathway involvement was observed across the different comparison groups, with carbohydrate metabolism consistently representing the largest proportion. For DEGs associated with lipid metabolism, the highest number was identified in the “L vs. R” comparison group, totaling 192 genes (Figure 1C). GO annotation of the hemp transcriptome data classified the DEGs into three main categories: cellular components (CCs), molecular functions (MFs), and biological processes (BPs) (Figure 1B). Within the cellular component category, 8987 DEGs were annotated, with the top five subcategories being integral components of membranes (2126 genes, 24%), plasma membranes (1383 genes, 15%), chloroplasts (1208 genes, 13%), membranes (531 genes, 6%), and chloroplast stromata (405 genes, 5%). In the molecular function category, 8250 DEGs were identified. The most represented subcategories included DNA-binding transcription factor activity (790 genes, 10%), sequence-specific DNA binding (428 genes, 5%), heme binding (235 genes, 3%), structural constituents of ribosomes (225 genes, 3%), and oxidoreductase activity (189 genes, 2%). As for biological processes, 8030 DEGs were annotated. The major subcategories were the defense response (363 genes, 5%), the response to salt stress (299 genes, 4%), and translation (191 genes, 2%).

### 3.3. Lipid Metabolism-Related Genes

**Figure 2:**
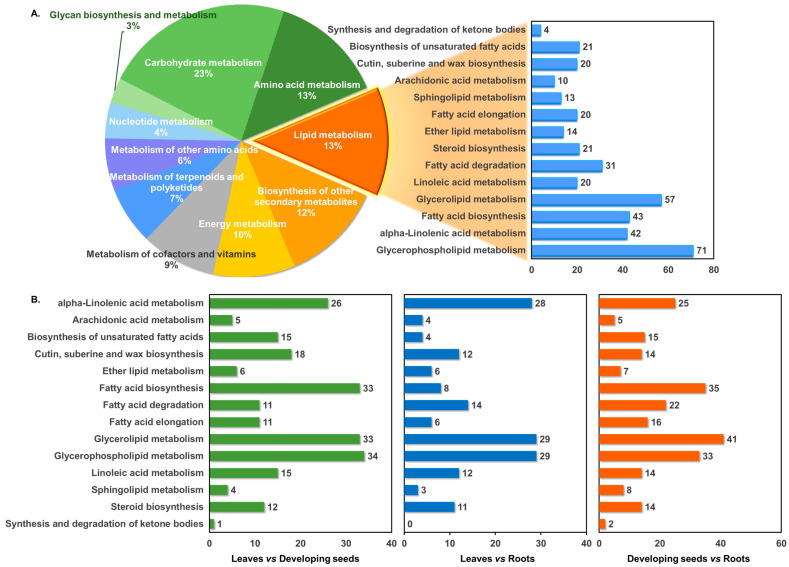
KEGG function classification and lipid-metabolism pathway enrichment analysis. (**A**) Distribution of metabolism-related DEGs annotated in the hemp transcriptome, with each color representing a different metabolic pathway. (**B**) DEGs associated with lipid-metabolism pathways across different tissue comparisons. Distinct colors indicate different comparison groups.

Based on the annotation results of the hemp transcriptome data, further analysis of the gene annotation associated with lipid metabolism found that DEGs related to lipid metabolism were mapped to fourteen lipid-metabolism pathways (full list and detailed information are listed in Supplementary Table S4). Among these, the most enriched pathways were glycerophospholipid metabolism, glycerolipid metabolism, and fatty-acid biosynthesis, with 71, 57, and 43 DEGs, respectively, representing 24%, 20%, and 15% of the lipid metabolism-related DEGs (Figure 2A). Tissue-specific pairwise comparisons revealed that the “S vs. R” group exhibited the highest number of lipid metabolism-related DEGs, totaling 192, of which 41 were involved in glycerolipid metabolism (21%). In the “S vs. L” group, 169 DEGs were annotated, including 34 related to glycerophospholipid metabolism (20%). The “L vs. R” comparison revealed 121 DEGs, with 29 associated with glycerolipid metabolism (23%) (Figure 2B). We found that across all tissue comparisons, glycerophospholipid metabolism, glycerolipid metabolism, and fatty-acid biosynthesis consistently showed high numbers and proportions of annotated DEGs (Figure 2B). Notably, in comparisons involving developing seeds and root tissues, glycerophospholipid- and glycerolipid-metabolism pathways exhibited the highest DEG representation.

To further investigate the regulatory patterns of lipid metabolism in hemp, genome-wide screening of lipid-related genes was conducted based on previously published studies [23,25]. Differentially expressed genes (DEGs) associated with lipid metabolism were identified across various hemp tissues using a threshold of |Log2FC| ≥ 1. Transcriptome analysis revealed a total of 696 genes involved in lipid metabolic pathways (Figure S2 and Table S5). Among the tissue comparisons, the “S vs. R” group exhibited the highest number of lipid-related DEGs, with 489 genes identified, 302 of which were upregulated, significantly outnumbering the 187 downregulated genes. In the “L vs. R” comparison, 341 lipid-related DEGs were detected, including 185 upregulated and 156 downregulated genes. The “L vs. S” group showed the lowest number of DEGs, with 304 genes, nearly equally divided between upregulated (153) and downregulated (151) genes. Most genes associated with fatty acids and photosynthetic-membrane lipid synthesis are highly expressed in leaf tissues, and genes associated with TAGs are highly expressed in developing seeds.

#### 3.3.1. Genes Associated with Triglyceride Biosynthesis

**Figure 3:**
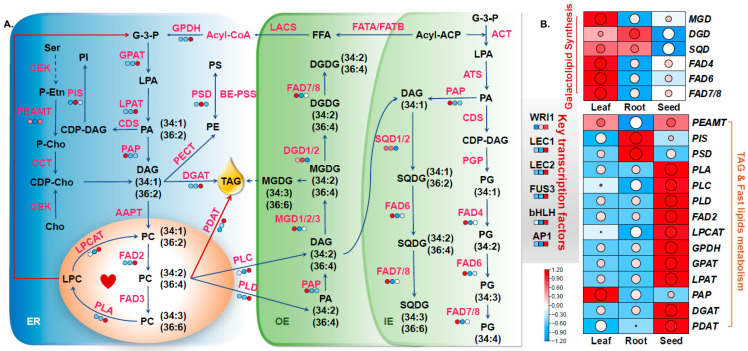
Schematic representation of lipid metabolism in hemp. (**A**) Visualization of the TAG and photosynthetic-membrane lipid metabolic regulatory network. Gene expression data were integrated into the corresponding metabolic pathways, with differential expression levels of key lipid-related genes illustrated as heat maps. The colors indicate FPKM values across different tissues, representing variations in gene expression and corresponding lipid metabolite types. (**B**) Heat map showing the expression profiles of genes involved in lipid biosynthesis pathways in hemp. The color gradient reflects expression intensity, with red indicating high expression and blue indicating low expression.

Triacylglycerol (TAG) serves as the primary storage form of oil in hemp, making the TAG biosynthetic pathway critical for oil accumulation. To elucidate the regulatory mechanisms of TAG biosynthesis across different tissues, differentially expressed genes (DEGs) involved in the TAG metabolic pathway were identified, including GPDH (glycerol-3-phosphate dehydrogenase), GPAT (glycerol-3-phosphate acyltransferase), LPAT (lysophosphatidyl acyltransferase), PAP (phosphatidic acid phosphatase), *DGAT*, and *PDAT*. Three *GPDH* genes (LOC115710084, LOC115710085, LOC115698811) exhibited consistent upregulation in both the “S vs. L” and “S vs. R” comparisons, with LOC115698811 showing Log2FC values of 5.12 and 5.55, respectively. One GPAT gene (LOC115697313) was also significantly upregulated in seeds, with Log2FC values of 2.69 (“S vs. L”) and 3.23 (“S vs. R”). Strong induction of DGAT genes was observed in both comparisons, with WSD1 showing the highest expression levels and Log2FC values, of 11.53 and 11.12, respectively (Table S6). Additionally, an upregulated *PDAT* gene showed expression increases of 5.89 (“S vs. L”) and 2.38 (“S vs. R”). Furthermore, two *FAD2* genes (*LOC115718446*, *LOC115719000*) and two *FAD3* genes (*LOC115720710*, *LOC115697681*) were identified as upregulated in the seed tissue. Notably, the *FAD2* genes and *FAD3* (*LOC115720710*) displayed high expression levels, with Log2FC values exceeding 5 (Figure 3 and Table S6). In addition, five *SAD* genes were highly expressed in developing seeds (LOC115705664, LOC115705684, LOC115707488, LOC115698723, LOC115708035). Among these, LOC115705664 exhibited the most pronounced upregulation, with Log2FC values of 9.38 (“S vs. L”) and 7.49 (“S vs. R”). These expression patterns may contribute to the elevated polyunsaturated fatty-acid content observed in industrial hemp.

#### 3.3.2. Analysis of Genes Involved in Photosynthetic-Membrane Lipid Synthesis

Further screening identified DEGs involved in the biosynthesis of photosynthetic-membrane lipids, including *MGD* (monogalactosyldiacylglycerol synthase), *DGD* (digalactosyldiacylglycerol synthase), and *SQD* (sulfoquinovosyldiacylglycerol synthase), as well as *FAD4*, *FAD6*, and *FAD7/8*. Genes associated with galactolipid metabolism exhibited upregulated expression in both the “L vs. R” and “L vs. S” comparison groups. Specifically, *MGD* (*LOC115707728*) showed Log2FC values of 2.29 (“L vs. R”) and 1.29 (“L vs. S”); *DGD* (*LOC115698658*) had Log2FC values of 1.18 and 1.05, respectively; and *SQD* (*LOC115697608*) exhibited Log2FC values of 1.57 (“L vs. R”) and 1.39 (“L vs. S”). Additionally, *FAD4* (*LOC115698783*) was markedly upregulated in both comparisons, with Log2FC values of 7.64 and 3.41, respectively. *FAD6* (*LOC115725117*) also showed significant upregulation, with Log2FC values of 2.67 (“L vs. R”) and 1.78 (“L vs. S”). Similarly, *FAD7* was significantly upregulated in leaf tissues, with Log2FC values of 4.53 and 2.30 in the “L vs. R” and “L vs. S” comparisons, respectively (Figure 3 and Table S6). *FAD6* (*LOC115725117*) exhibited significant upregulation in both the “L vs. R” and “L vs. S” comparison groups, with Log2FC values of 2.67 and 1.78, respectively. Similarly, *FAD7* showed marked upregulation in the same comparisons, with Log2FC values of 4.53 and 2.30, respectively.

#### 3.3.3. Analysis of DEGs in Fatty-Acid Metabolic Pathways

**Figure 4:**
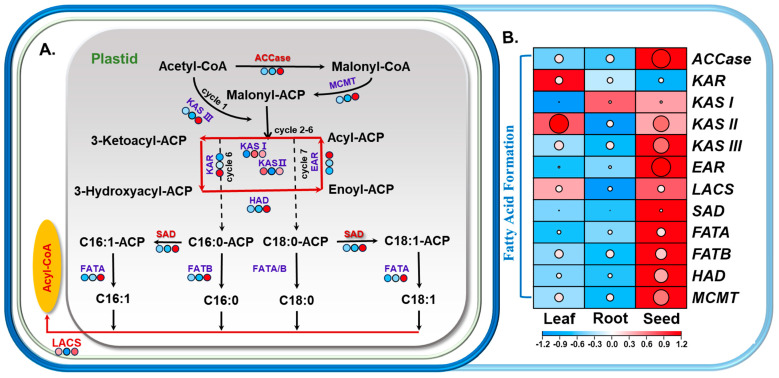
Transcriptomic profiling of lipid metabolism-related genes in the fatty-acid biosynthesis pathways of hemp. (**A**) Schematic representation of the fatty-acid biosynthesis pathway in hemp, with gene expression data overlaid onto the corresponding metabolic pathway diagram. Differential expression levels of key lipid-related genes are visualized as heat maps, indicating tissue-specific variations in gene activity and associated lipid metabolite types. The different colors were used to indicate the FPKM values of lipid metabolism genes in different tissues. (**B**) Heat map showing the expression patterns of genes involved in fatty-acid biosynthesis. The color gradient indicates expression levels, where red represents high expression and blue represents low expression.

Numerous DEGs related to fatty-acid synthase were identified in the hemp transcriptome. ACCase, a key rate-limiting enzyme in fatty-acid metabolism, was represented by one upregulated gene (*LOC115712810*) in both the “S vs. L” and “S vs. R” comparison groups, with Log2FC values exceeding 2 (Figure 4, Table S6). Genes encoding β-ketoacyl-ACP synthases (*KASI*, *KASII*, and *KASIII*) were expressed at notably higher levels in developing seeds compared to other tissues. In particular, two *KASII* genes (*LOC115707135* and *LOC115707136*) showed strong upregulation (Log2FC > 6). *KASIII* (*LOC115717112*), which catalyzes the initial step in fatty-acid elongation, also exhibited upregulated expression, with Log2FC values of 1.42 (“S vs. L”) and 1.96 (“S vs. R”). Five *SAD* genes were significantly upregulated in seed tissues (*LOC115705664*, *LOC115705684*, *LOC115707488*, *LOC115698723*, *LOC115708035*). Among these, *LOC115705664* showed the highest expression levels, with Log2FC values of 9.38 and 7.49 in the “S vs. L” and “S vs. R” groups, respectively, potentially contributing to the high content of unsaturated fatty acids in hemp. Additionally, *FATA* and *FATB* thioesterase genes were upregulated in both tissue comparisons, with *FATA* (*LOC115714069*) showing particularly elevated expression (Log2FC = 2.17 in “S vs. L” and 1.97 in “S vs. R”), suggesting a role in promoting the accumulation of unsaturated fatty acids (Figure 4, Table S6).

### 3.4. Lipidome of Hemp

**Figure 5:**
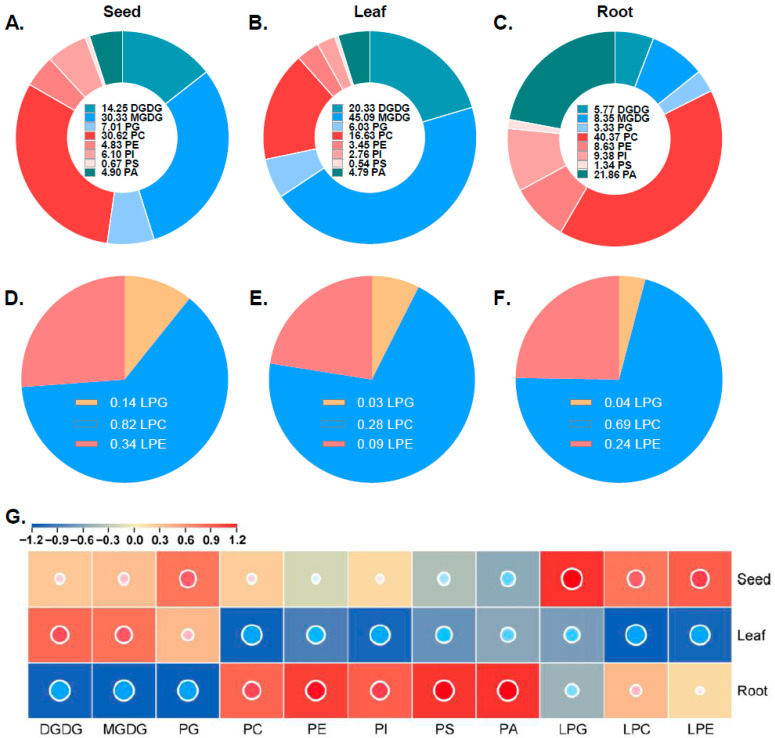
The concentrations of each lipid class of hemp in different tissues. Pie charts illustrating the proportional distributions of major lipid classes in developing seeds (**A**), leaves (**B**), and roots (**C**) of hemp. (**D**–**F**) Pie charts showing the distribution of lysophospholipid subclasses in seeds (**D**), leaves (**E**), and roots (**F**). Distinct colors represent different lipid classes. (**G**) Heat map displaying the relative concentration of each lipid class across hemp tissues. The color gradient indicates the concentration levels, with red denoting high abundance and blue indicating low abundance.

Lipidomic analysis was conducted to profile the major lipid classes and fatty-acid composition in hemp leaves, developing seeds, and root tissues. A total of 11 lipid components were identified, including two galactolipids (MGDG, DGDG); six phospholipids, including PC, PI, PE, PS, PA, and PG; and 3 lysophospholipids, including LPA, LPC, and LPG (Figure 5). In developing seed tissues, PC was the most abundant phospholipid, comprising 30.6% of the total lipid content. Galactolipids collectively accounted for 44.6%, with MGDG representing 68% of this category, making it the dominant galactolipid. Lysophospholipids were present at 1.3%, a higher proportion than in leaf or root tissues. In leaf tissues, galactolipids (MGDG and DGDG) were predominant, accounting for 65.4% of the total lipids, with MGDG alone contributing nearly 50%. Phospholipids represented 34.2% of the total lipids, with PC being the most abundant at 16.6%. Other lipid components were detected at lower levels. The elevated levels of galactolipids and PG, key constituents of the photosynthetic membrane, suggest their important role in photosynthesis. In contrast, root tissues showed the highest phospholipid content, comprising 84.9% of the total lipids. PC was the most abundant at 40.4%, followed by PA at 21.9%. In comparison, galactolipids and PG were present at much lower levels, at 14.1% and 3.3%, respectively, indicating a reduced role of photosynthetic-membrane lipids in roots.

### 3.5. Analysis of Molecular Species Types of Lipids in Different Tissue Sites

**Figure 6:**
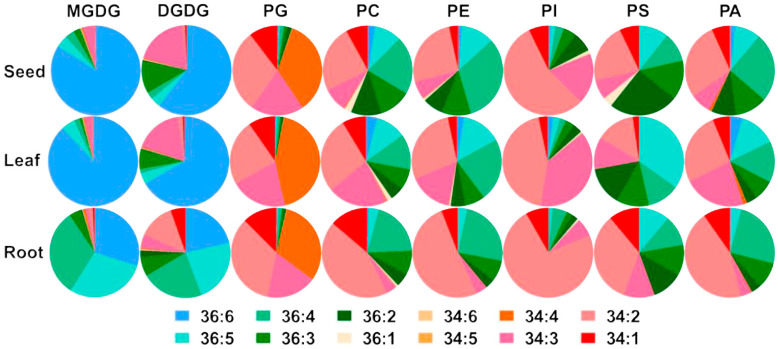
The distributions of glycerolipid and phospholipid lipid molecular species in the different tissues of hemp. This chart illustrates the compositions of various lipid molecular species in hemp leaves, seeds, and roots. Different colors represent distinct lipid molecular species.

In addition to profiling overall lipid classes, the molecular species of the major lipid-associated fatty-acid side chains in hemp were analyzed. The predominant lipid molecular species in hemp were found to be C34 and C36, with galactolipids mainly consisting of C36 species—particularly C36:6, which emerged as the most abundant subtype (Figure 6). Within MGDG, the molar percentage of C36:6 in leaf tissue reached as high as 88%, significantly higher than in seeds or roots. For DGDG, the dominant molecular species in seeds and leaves were C34:3 and C36:6, while in the roots, C36:5, C36:4, C34:2, and C34:1 were more prevalent. Overall, the predominance of C36:6 in both MGDG and DGDG across tissues suggests that these galactolipids are primarily synthesized via the eukaryotic (endoplasmic reticulum-derived) pathway, indicating that hemp is a typical 18:3 plant. The phospholipid species in hemp were also dominated by C34 and C36 molecules, with C34:2 and C36:4 being the most abundant. In PG, C34 was the primary species, with C34:4, C34:3, and C34:2 dominating across tissues. In PC, C36:4 and C34:2 were the major species in developing seeds and roots, while C34:2 was predominant in leaves. The molecular profile of PE closely resembled that of PC, with C36:4 and C34:2 being most abundant in developing seeds. For PI, the composition was similar to that of PG, with C34 species predominating, especially C34:2 and C34:3. C34:2 was particularly dominant in seeds and root tissues. PS, though present in lower amounts, was enriched in the C36:5 species across tissues compared to other lipid classes. In PA, C34:2 and C36:4 were predominant in seeds and roots, while C34:2, C34:3, C36:4, and C36:5 were the major molecular species in leaves.

## 4. Discussion

Currently, in comparison to major staple crops, hemp has relatively limited genetic resources and molecular biology research. In this study, we generated transcriptomic data from various hemp tissues using RNA-seq, yielding a total of 60.7 GB of high-quality transcriptome data. This dataset was used to explore the regulatory mechanisms governing the genes involved in lipid-metabolism pathways. The identified DEGs in hemp span a broad range of biological processes and metabolic pathways and include many with known functions related to lipid metabolism. The tissue-specific distribution of lipid classes follows a conservative pattern across diverse plant species, including rice, tomato, maize, and sorghum; glycolipids are markedly enriched in photosynthetic leaf tissues, whereas phospholipids predominate in root systems [26,27,28,29]. By integrating transcriptomic and lipid metabolomic analysis across different hemp tissues, we constructed a proposed molecular model of lipid metabolism (Figure 3 and Figure 4).

**Figure 7:**
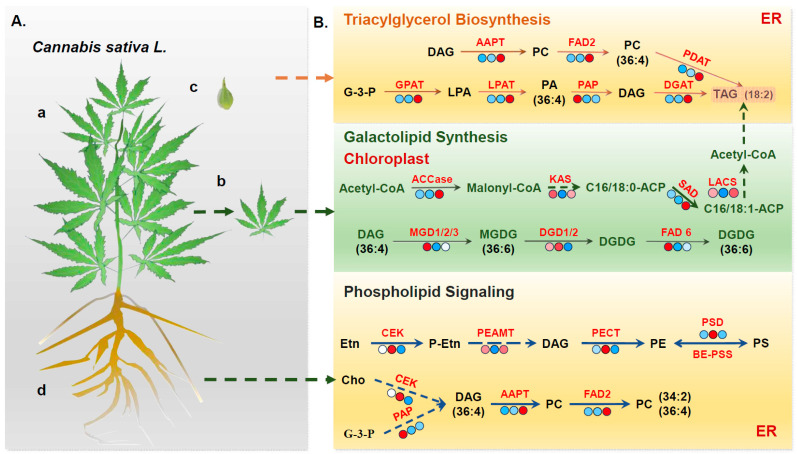
Proposed model for hemp lipid metabolism pattern. (**A**) Schematic representations of hemp plant structure and sampled tissues: (a) whole hemp plant, (b) leaf tissue, (c) developing seed tissue, and (d) root tissue. (**B**) Transcriptomic analysis reveals distinct expression patterns of lipid-related genes across different tissues. A heatmap displays the expression levels of genes involved in key lipid biosynthesis pathways, with varying colors indicating differential expression. This provides a clear visual comparison of tissue-specific gene activity related to lipid metabolism.

In developing seeds of hemp, genes associated with TAG were found to be positively expressed (Figure 7). GPAT, a central enzyme linking fatty-acid and glycerolipid biosynthesis, catalyzes the key rate-limiting initial step of glycerolipid biosynthesis and has been shown to play a critical role in oil biosynthesis in various plant species [30,31,32]). In this study, the gene encoding *GPAT* was markedly upregulated in developing seeds, suggesting that *GPAT* is crucial for oil accumulation during hemp seed development. The LPAT catalyzes the second acylation step of the Kennedy pathway, converting LPA to PA. PA is subsequently converted to DAG and finally to TAG under the catalysis of DGAT [33]. In general, DGAT1 is broadly involved in TAG biosynthesis in most plant species, while DGAT2 is more specialized in accumulating unusual fatty acids, such as DHA and EPA [34,35], and DGAT expression affects seed development and oil content [34,36]. In the present study, one LPAT gene and five DGAT genes were identified as significantly upregulated in developing hemp seeds (Table S6), further supporting their functional relevance in hemp oil biosynthesis and accumulation. PC plays a pivotal role in lipid metabolism in plant cells. It not only serves as a major structural component of cellular membranes but also acts as a crucial acyl donor for lipid biosynthesis and an indispensable substrate for fatty-acid modifications, including desaturation and hydroxylation [37]. The “Lands cycle”, a key pathway for rapid PC turnover and acyl remodeling, is mainly mediated by enzymes such as FAD2, PLA, and LPCAT [38]. In the hemp transcriptome, simultaneous upregulation of *FAD2*, *PDAT*, and *LPA* contributes to the accumulation of 36:4 PC and 18: 2 TAGs, which may be an important reason for the high unsaturated fatty-acid content in hemp seeds.

Leaves, serving as the primary site for fatty-acid biosynthesis, exhibit an intimate association between their lipid metabolism and photosynthetic processes. In plant chloroplasts, the gene encoding *ACCase* was found to be significantly upregulated in developing seeds compared to other tissues, indicating its central role in supplying fatty-acid precursors for oil production. This observation is consistent with previous studies demonstrating that overexpression of *ACCase* significantly increases oil content in *Chlamydomonas reinhardtii* [39]. KAS, specifically KASI, KASII, and KASIII, is critical for catalyzing condensation reactions during fatty-acid chain elongation [40]. In the current study, genes encoding KAS were generally upregulated in both leaves and developing seeds of hemp, suggesting a prominent role of *KAS* in driving fatty-acid biosynthesis in these tissues. Hemp seeds are particularly rich in unsaturated fatty acids, which can comprise over 90% of the total seed oil content and which are recognized for their significant nutritional and health benefits [41]. Fatty-acid desaturation predominantly takes place in the chloroplasts, where the rate-limiting enzyme SAD catalyzes the conversion of stearoyl-ACP to oleoyl-ACP. The critical function of SAD in unsaturated fatty-acid biosynthesis has been well-documented in several oil-rich crops [42,43,44]. In this study, transcriptome analysis revealed that the *SAD* gene was significantly upregulated in developing hemp seeds, which could correspond to the accumulation of unsaturated fatty acids in hemp. MGDG and DGDG, primary constituents of photosynthetic-membrane lipids, are essential for chloroplast membrane integrity and photosynthetic efficiency [45]. Galactolipid biosynthesis occurs via two distinct pathways: the prokaryotic pathway and the eukaryotic pathway [46]. For instance, in species like *Arabidopsis thaliana* and spinach, both pathways contribute to the synthesis of MGDG and DGDG, resulting in a prominent presence of C16 fatty acids, which are known as”16:3 plants”. In contrast, plants such as maize and wheat predominantly utilize the eukaryotic pathway, which leads to C36:6 becoming the dominant molecular species in their galactolipids, categorizing them as “18:3 plants” [47,48]. In the present study, C36:6 was identified as the major galactolipid species in hemp leaves, indicating that hemp is a typical 18:3 plant. Supporting this, transcriptome analysis revealed that several genes encoding *MGD* and *DGD* were significantly upregulated in leaf tissues compared to other plant parts (Figure 3 and Table S6), further highlighting the active galactolipid biosynthesis in photosynthetically active tissues of hemp.

Lipid metabolism in root tissue focuses on phospholipid accumulation and adverse responses. Genes closely related to phospholipid biosynthetic pathways, such as *PSD*, *PECT*, and *AAPT*, showed increased expression in the root tissues. Consistently, a greater accumulation of phospholipids was detected in roots relative to other tissues. Previous studies have shown that phospholipid metabolism-related genes play essential roles in plant responses to abiotic stresses [38,49]. Thus, given that the roots are the primary organ in direct contact with the soil environment, the enrichment of phospholipids and the root-specific expression of these metabolic genes may contribute to the resilience of hemp to various environmental stressors, such as salinity, heavy metals, and alkaline conditions.

In this study, an integrated transcriptomic and lipidomic analysis was conducted to investigate the regulatory patterns of lipid metabolism in different tissues of hemp. This study revealed that the composition and relative abundance of lipid species in hemp exhibit strong tissue specificity. Importantly, the expression patterns of genes involved in the biosynthesis and regulation of these lipid components also displayed a corresponding tissue-specific trend. Genes associated with the metabolism of lipid classes, such as glycerolipids, phospholipids, and galactolipids, were differentially expressed in tissues where those lipid types were enriched. These findings strongly suggest that the observed tissue-specific lipid distribution in hemp is driven by differential gene expression and the activity of key enzymes within lipid metabolic pathways. Elucidating the precise identity of these coding genes and their cognate metabolic pathways will furnish essential genetic resources and mechanistic insights for the targeted, genome-edited refinement of crop lipid profiles. Furthermore, this study integrates multi-tissue transcriptomic and lipidomic data to systematically characterize the tissue-specific regulatory network governing lipid synthesis in hemp, providing a verifiable theoretical framework for the “gene-metabolite-phenotype” association. Building on these findings, future work can further explore latent regulatory factors and their functional variants by combining genome-wide association studies with multi-omics modeling. Additionally, synthetic biology approaches can be employed to construct high-yield, high-specificity lipid cell factories, facilitating the paradigm shift from empirical breeding to design-based breeding in hemp. These strategies not only lay the theoretical and technical groundwork for the high-value utilization of this species in the food, pharmaceutical, and bioenergy sectors but also offer a transferable methodological pathway for lipid engineering in other plants.

## 5. Conclusions

In conclusion, this study conducted transcriptomic and lipidomic integration analysis on developed hemp seeds, leaves, and root tissues. The results revealed that the DEGs associated with lipid metabolism were primarily enriched in glycerophospholipid metabolism, glycerolipid metabolism, and fatty-acid biosynthesis. In parallel, lipidomic profiling identified 11 distinct lipid classes across the tissues. Leaves were dominated by galactolipids, accounting for 65.4% of the total lipids, with C36:6 as the predominant species—consistent with the characteristic “18:3 plant” lipid profile. The roots showed a high abundance of phospholipids, representing 84.9% of their lipid composition. The seeds, meanwhile, were rich in PC and galactolipids. Transcriptomic analysis revealed that the lipid composition of hemp seeds is closely linked to the expression of genes involved in lipid metabolism, exhibiting a clear tissue-specific expression pattern. Genes associated with TAG biosynthesis were predominantly and highly expressed in developing seeds, whereas genes involved in galactolipid synthesis were most abundant in leaf tissues and those related to phospholipid biosynthesis showed the highest expression in root tissues. *SAD*, *FAD2*, and *FAD3* show high expression levels in developing seeds. This likely contributes to the cumulation of unsaturated fatty acids, particularly PUFAs, in hemp. The findings of this study offer valuable insights into the molecular regulation of lipid metabolism in *C. sativa* and establish a theoretical foundation for future research. Furthermore, these results provide important genetic resources for the improvement of oil yield and quality in hemp and other oilseed crops through targeted breeding or biotechnological approaches.

## Data Availability

The original contributions presented in the study are included in the article/Supplementary Material, further inquiries can be directed to the corresponding author.

## Supplementary Materials

The following supporting information can be downloaded at: https://www.mdpi.com/article/10.3390/foods14162809/s1, Figure S1: Quantitative real-time PCR assay for lipid metabolism-related DEGs of hemp leaves. qRT-PCR analysis was performed to validate the expression levels of selected DEGs associated with lipid metabolism in hemp leaves; Figure S2: DEGs involved in metabolic pathways, with different colors representing different tissue comparison groups; Table S1: Primer sequences used for quantitative real-time PCR (qRT-PCR) analysis; Table S2: Statistics of RNA-Seq data of nine hemp samples; Table S3: The DEGs associated with various metabolism pathways; Table S4: Lipid metabolism pathways involving identified DEGs; Table S5: Tissue-specific DEGs related to lipid metabolism in *C. sativa*; Table S6: DEGs involved in major lipid metabolic pathways in *C. sativa*.

## Abbreviations

ACCase: acetyl-CoA carboxylase; ALA: α-linolenic acid; DAG: diacylglycerol; DEG: differentially expressed gene; DGAT: diacylglycerol acyltransferase; DGD: digalactosyldiacylglycerol synthase; DGDG: digalactosyldiacylglycerol; EFAs: essential fatty acids; ER: endoplasmic reticulum; FADs: fatty-acid desaturases; FAS: fatty-acid synthase complex; FPKM: fragments per kilobase of transcript per million mapped reads; GO: Gene Ontology; GPAT: glycerol-3-phosphate acyltransferase; GPDH: glycerol-3-phosphate dehydrogenase; KEGG: Kyoto Encyclopedia of Genes and Genomes; LA: linoleic acid; LPAT: lysophosphatidyl acyltransferase; MGD: monogalactosyl-diacylglycerol synthase; MGDG: monogalactosyldiacylglycerol; PA: phosphatidic acid; PAP: phosphatidic acid phosphatase; PC: Phosphatidylcholine; PDAT: phospholipid diacylglycerol acyltransferase; PE: phosphatidylethanolamine; PG: phosphatidylglycerol; PI: phosphatidylinositol; PLA: Phospholipase A; PLC: Phospholipase C; PLD: Phospholipase D.; PS: phosphatidylserine; PUFAs: polyunsaturated fatty acids; QRT: real-time PCR; SAD: stearoyl-acyl carrier protein desaturase; SQD: sulfoquinovosyldiacylglycerol synthase; SQDG: sulfoquinovosyldiacylglycerol; TAG: triacylglycerol; THC: tetrahydrocannabinol.
