# Supplementary figures and images for: Transcriptomic and Lipidomic Analysis Reveals the Regulatory Network of Lipid Metabolism in Cannabis sativa
Source: Foods. 2025 Aug 13;14(16):2809. doi: 10.3390/foods14162809 (PMC12385353; doi:10.3390/foods14162809)

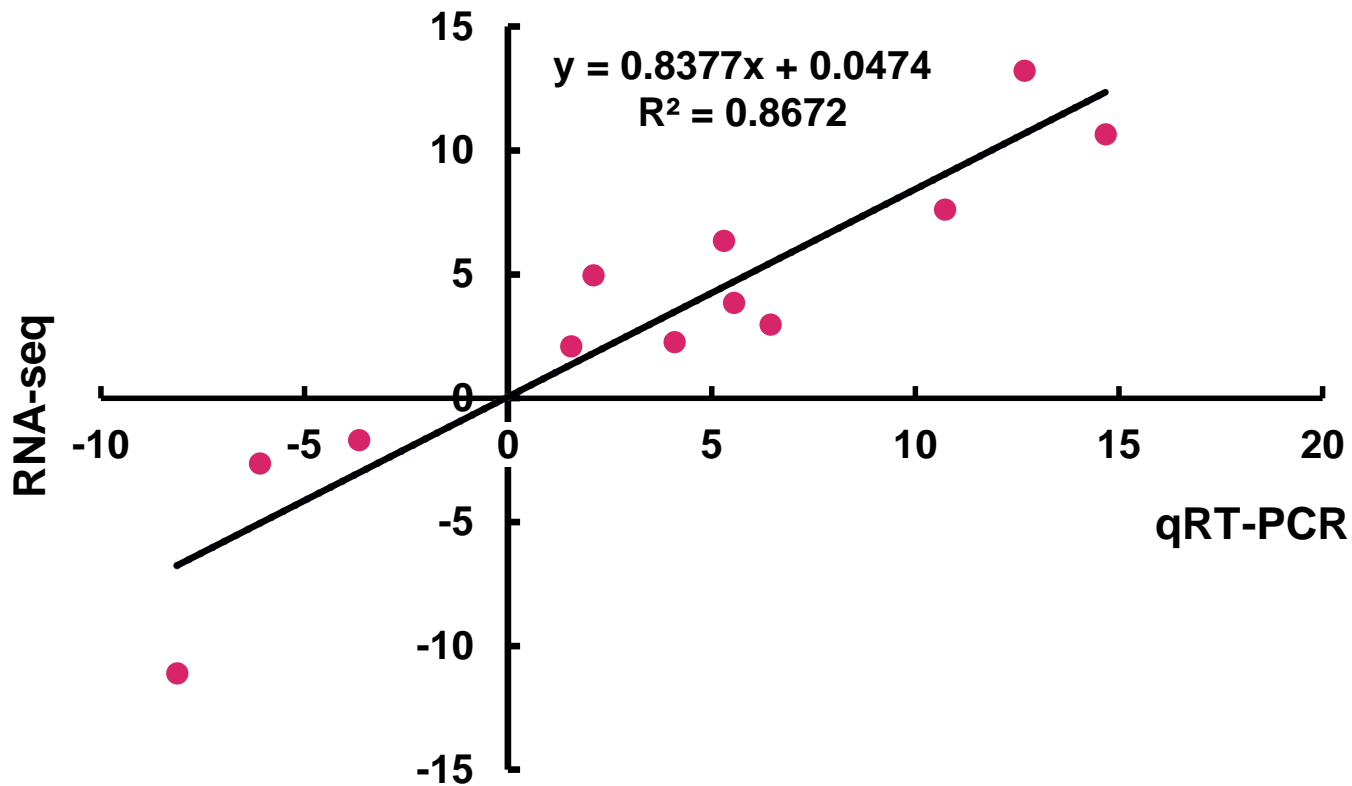

Supplement: Supplementary file 1 [file foods-14-02809-s001.zip › Figure S1.pdf]

Differentially expressed genes related to  
lipid metabolism in *C. sativa*

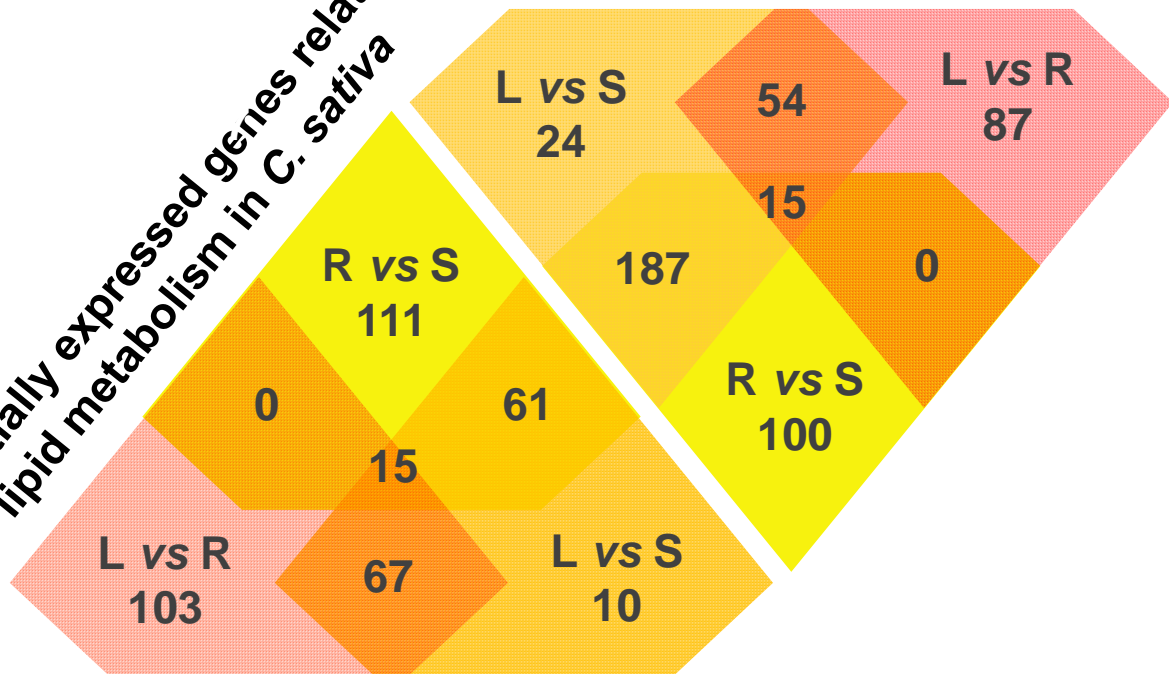

Supplement: Supplementary file 1 [file foods-14-02809-s001.zip › Figure S2.pdf]
